# Genome-wide association studies for canine hip dysplasia in single and multiple populations – implications and potential novel risk loci

**DOI:** 10.1186/s12864-021-07945-z

**Published:** 2021-09-02

**Authors:** Shizhi Wang, Erling Strandberg, Per Arvelius, Dylan N. Clements, Pamela Wiener, Juliane Friedrich

**Affiliations:** 1 Division of Genetics and Genomics, The Roslin Institute and Royal (Dick) School of Veterinary Studies, University of Edinburgh, Midlothian, EH25 9RG UK; 2 Department of Animal Breeding and Genetics, Swedish University of Agricultural Sciences, PO Box 7023, S-750 07 Uppsala, Sweden; 3 Swedish Armed Forces Dog Training Centre, Box 194, SE-195 24, Märsta, Sweden; 4 Royal (Dick) School of Veterinary Studies, University of Edinburgh, Midlothian, EH25 9RG UK

**Keywords:** German shepherd dogs, FCI grades, BVA/KC scores, QTL, Genetic diversity

## Abstract

**Background:**

Association mapping studies of quantitative trait loci (QTL) for canine hip dysplasia (CHD) can contribute to the understanding of the genetic background of this common and debilitating disease and might contribute to its genetic improvement. The power of association studies for CHD is limited by relatively small sample numbers for CHD records within countries, suggesting potential benefits of joining data across countries. However, this is complicated due to the use of different scoring systems across countries. In this study, we incorporated routinely assessed CHD records and genotype data of German Shepherd dogs from two countries (UK and Sweden) to perform genome-wide association studies (GWAS) within populations using different variations of CHD phenotypes. As phenotypes, dogs were either classified into cases and controls based on the *Fédération Cynologique Internationale* (FCI) five-level grading of the worst hip or the FCI grade was treated as an ordinal trait. In a subsequent meta-analysis, we added publicly available data from a Finnish population and performed the GWAS across all populations. Genetic associations for the CHD phenotypes were evaluated in a linear mixed model using 62,089 SNPs.

**Results:**

Multiple SNPs with genome-wide significant and suggestive associations were detected in single-population GWAS and the meta-analysis. Few of these SNPs overlapped between populations or between single-population GWAS and the meta-analysis, suggesting that many CHD-related QTL are population-specific. More significant or suggestive SNPs were identified when FCI grades were used as phenotypes in comparison to the case-control approach. *MED13* (Chr 9) and *PLEKHA7* (Chr 21) emerged as novel positional candidate genes associated with hip dysplasia.

**Conclusions:**

Our findings confirm the complex genetic nature of hip dysplasia in dogs, with multiple loci associated with the trait, most of which are population-specific. Routinely assessed CHD information collected across countries provide an opportunity to increase sample sizes and statistical power for association studies. While the lack of standardisation of CHD assessment schemes across countries poses a challenge, we showed that conversion of traits can be utilised to overcome this obstacle.

**Supplementary Information:**

The online version contains supplementary material available at 10.1186/s12864-021-07945-z.

## Background

Canine hip dysplasia (CHD), a condition involving abnormal development of the coxofemoral (hip) joint, is one of the most common orthopaedic disorders in dogs and has been reported for more than 177 dog breeds, with a prevalence varying from 0.9 to 75.3%, according to the Orthopedic Foundation for Animals (OFA) [1]. CHD can lead to lameness, arthritis and hip pain and thus has a profound effect on animal welfare. CHD is recognised as a heritable disease, and several studies aimed to identify underlying genetic risk factors [2–4]. The inheritance pattern of CHD has been shown to be polygenic in GSDs [5] and regional and chromosomal heritability analyses of CHD in Labrador retrievers also indicated that the genetic architecture of CHD is based on many genes with small or moderate effect [4]. Identification of potential risk loci involved in CHD and a better understanding of the genetic architecture of this disease could help to address the severe welfare consequences, e.g. by promoting early genetic screening and improving breeding schemes [6, 7].

In a previous genome-wide association study (GWAS) on CHD and hip osteoarthritis in 721 dogs from eight breeds, SNPs associated with CHD were detected, suggesting several positional candidate genes [3]. Other studies using GWAS for CHD have not detected consistent genomic positions of quantitative trait loci (QTL) [4, 8–11], which might be explained by differing features across studies, including sample sizes, the CHD-related trait used as phenotype and population structure, in addition to different breeds. Merging CHD records and genotype data across countries might improve the statistical power of GWAS by increasing the currently limited sample size of genotyped animals within countries. Furthermore, the use of multiple populations, and thus increased diversity, might also enable the detection of further QTL for this complex disease, as shown in studies of human complex traits [12]. However, CHD breeding and assessment schemes vary between countries; the OFA screening system is used in the United States and Canada, while most European countries record CHD using the five-grade approach proposed by the FCI (*Fédération Cynologique Internationale*) and others measure the severity of CHD using hip scores established by the BVA/KC (*British Veterinary Association/Kennel Club*) (e.g. UK, Australia) [13]. The challenges and caveats of analysing CHD data from different countries have been previously addressed in cross-country genetic evaluation studies of CHD [14] in which conversion of BVA/KC scores into FCI grades slightly improved prediction accuracy of estimated breeding values for CHD, thus indicating the potential benefits of such approaches.

In the current study, we combined CHD phenotype and genotype data from British and Swedish German Shepherd dogs (GSDs) with publicly available data from a Finnish GSD population [11] to study the genetic architecture of CHD. We performed both single-population genome-wide association studies (GWAS) and a meta-analysis (GWAS across populations), with two aims: (I) to compare the consistency of results from different datasets and the performance of different phenotype classifications and (II) to identify potential novel loci influencing CHD by utilising multiple populations.

## Results

### Population structure

**Fig. 1:**
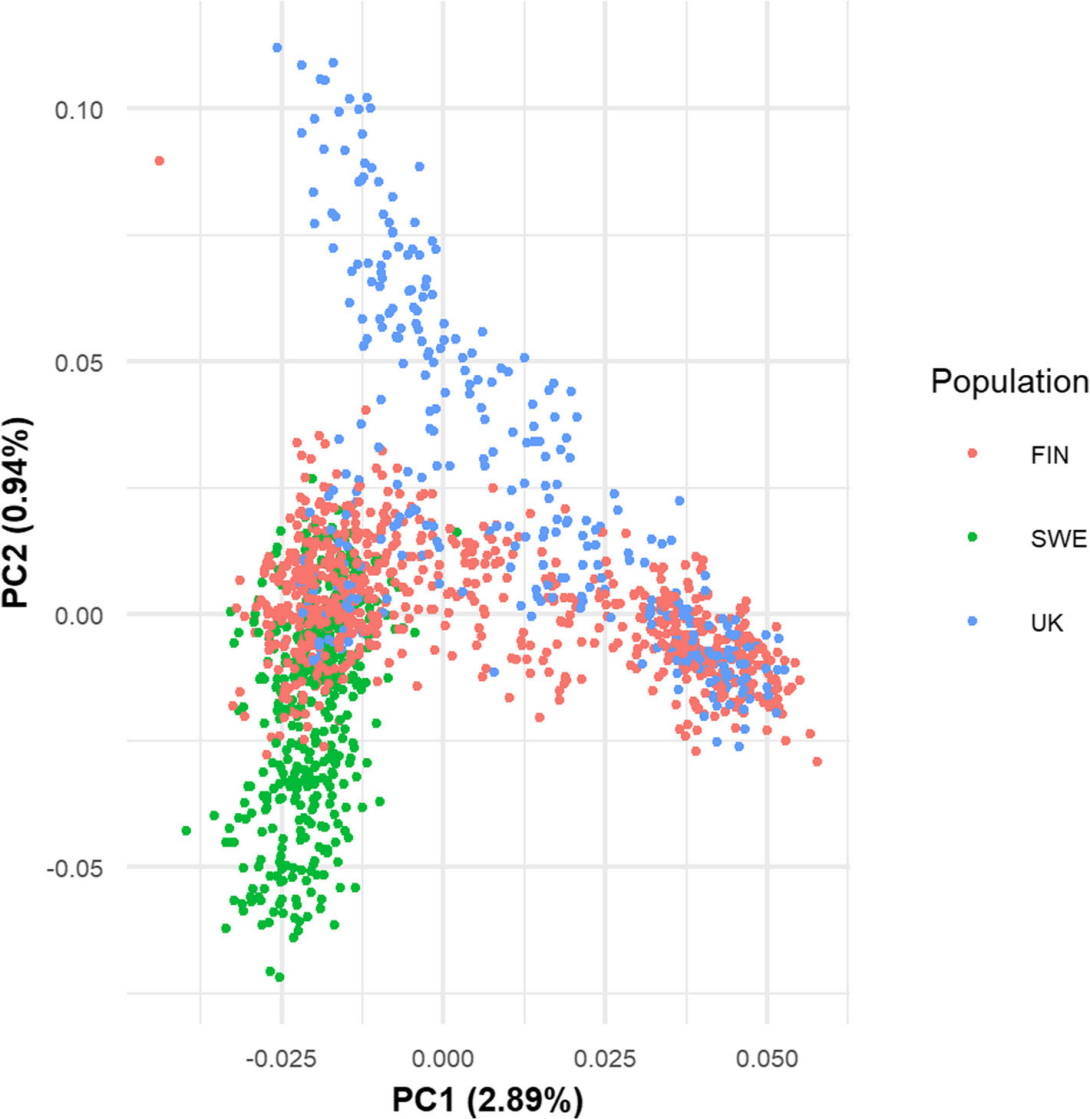
Principal component analysis for meta-analysis of the pruned genomic data. Eigenvalues for the first two principal components are plotted and dogs are coloured according to their population

The underlying genetic population structure of GSDs (from UK, Sweden and Finland) within and across populations was examined by PCA using a pruned genotype data set (comprising 5167 variants). In the multi-population PCA, including the UK, Swedish and Finnish dogs analysed for the meta-analysis, the first two PCs explained 2.89 and 0.94% of the variance, respectively. Figure 1 shows that overlaps exist between the populations, but a separation of clusters for the three countries is also visible, particularly associated with PC2.

### Association mapping

The associations between 62,089 SNPs and CHD records within populations and in a subsequent meta-analysis across populations was analysed by genome-wide association studies (GWAS). We took two approaches, the first was based on hip scores and the second was a case-control analysis, where dogs with the lowest scores (FCI-A or BVA/KC score 0–10) were treated as controls and all other dogs were considered cases. The quantile-quantile (QQ) plots (Fig. S1) and genomic inflation factor (λ) (ranging from 0.96 to 1.03), indicate that both within-population and meta-analysis GWAS were adequately controlled for population stratification.

FCI grades for the worse hip were used as phenotypes for the within-population GWAS of Swedish and Finnish dogs. For the UK dogs, the hip status was screened by the BVA/KC scheme, where aggregated scores for bilateral joints (total hip score; HS) are given. To enable a combined analysis with Swedish and Finnish FCI grades in a meta-analysis, we transformed UK HS to FCI grades. UK single-population GWAS were performed for both HS and FCI grades. The results were highly similar (e.g. more than half of the top 1% significant SNPs overlapped) and therefore the results for UK HS are presented in the supplement (Fig. S2, Table S1).

**Fig. 2:**
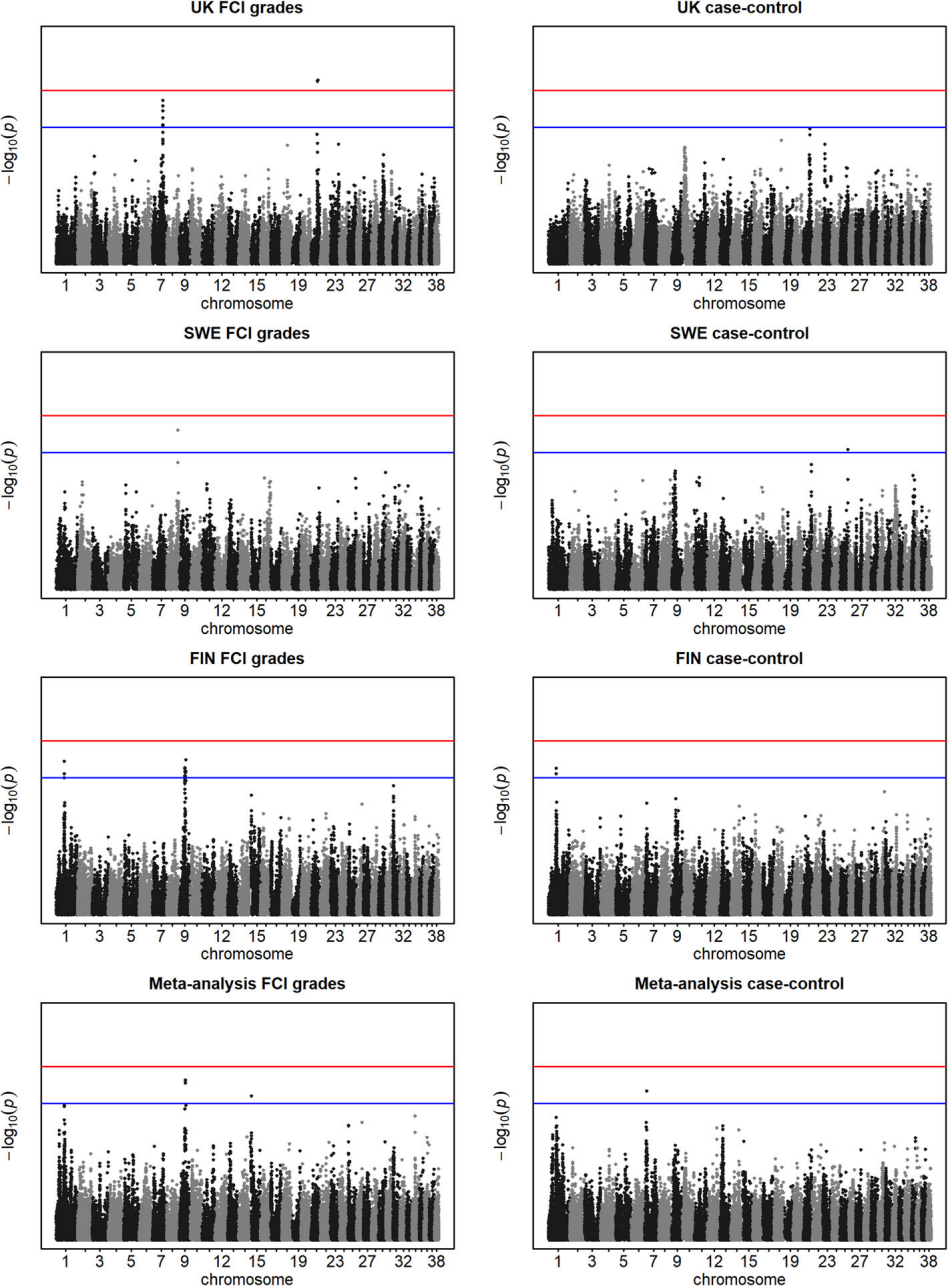
Manhattan plots for all CHD phenotype approaches in single-population and meta-analysis GWAS. The Finnish-population and meta-analysis GWAS are based on data provided by Mikkola et al. [11] and the Finnish-population GWAS is a repetition of the original GWAS with altered phenotypes. Manhattan plots were produced for the GWAS in UK (*n* = 180), Swedish (*n* = 402) and Finnish (*n* = 775) German Shepherd dog populations and for the meta-analysis GWAS (*n* = 1357). Genome-wide significance level is indicated by the red line and a suggestive association by the blue line

Five genome-wide significant associations were identified for the UK FCI approach on Chr 21 (39.2–39.4 Mbp) (Table 1, Fig. 2). In addition, across all within-population and meta-analyses, 32 suggestive associations (allowing one false-positive per genome scan) were identified, with most of them exclusively associated with a single population and phenotype approach (Table 1). Direct overlaps (shared SNPs) were only found between the FCI and case-control approaches for the Finnish population (on Chr 1) and between the FCI approach in the Finnish and meta-analysis GWAS (on Chr 9). Repeating the GWAS for the Finnish population [11] with altered phenotypes, regions with suggestive associations to CHD were detected on Chr 1 (45.1–46.6 Mbp) and on Chr 9 (31.3–36.8 Mbp). For the UK population, in addition to the genome-wide significant region on Chr 21, a region with suggestive association to FCI was detected on Chr 7 (58.5–58.8 Mbp). For the Swedish population, only single SNPs on Chr 8 and 25 showed suggestive associations with CHD. In the meta-analysis GWAS, for the FCI grade approach, the region on Chr 9 was detected (in the same region as the Finnish FCI grade GWAS), while for the case-control approach one SNP showed suggestive association on Chr 7, 5 Mbp upstream from the region showing a suggestive association in the UK population.

**Table 1:** SNPs with suggestive association identified within all CHD phenotype approaches in single-population and meta-analysis GWAS. GWAS were performed for 180 (UK), 402 (Swedish), 775 dogs (Finnish) and 1357 (meta-analysis) dogs, respectively. The Finnish-population and meta-analysis GWAS are based on data provided by Mikkola et al. [11] and the Finnish-population GWAS is a repetition of the original GWAS with altered phenotypes (see Materials and methods for further details)

SNP ID	Chr	Pos (bp)	AF^$^	β ± SE	p-value	Trait	Gene(s)^§^
BICF2S23248027	1	45,161,186	0.39	0.13 ± 0.03	1.14E-05	FIN case-control	*TIAM2, TFB1M, CLDN20,****NOX3***
BICF2S23248027	1	45,161,186	0.39	0.38 ± 0.09	1.17E-05	FIN-FCI	*TIAM2, TFB1M, CLDN20,****NOX3***
BICF2P468585	1	45,382,633	0.39	0.13 ± 0.03	7.28E-06	FIN case-control	NOX3
BICF2P468585	1	45,382,633	0.39	0.40 ± 0.09	4.21E-06	FIN-FCI	NOX3
BICF2P1037296	1	46,268,586	0.42	0.38 ± 0.09	1.56E-05	FIN-FCI	*ARID1B*
BICF2S22930063	7	11,089,390	0.20	−0.11 ± 0.03	5.96E-06	Meta-analysis case-control	*NSL1, TATDN3, ENSCAFG00000029517, FLVCR1,****VASH2****, ANGEL2, RPS6KC1*
BICF2G630561445	7	58,512,418	0.15	0.63 ± 0.13	2.85E-06	UK-FCI	*DSC1, DSC2, DSC3*
BICF2G630561553	7	58,588,983	0.26	0.54 ± 0.11	1.86E-06	UK-FCI	*DSC2, DSC3*
BICF2P566919	7	58,676,276	0.16	0.59 ± 0.13	1.44E-05	UK-FCI	
BICF2G630561779	7	58,791,059	0.15	0.63 ± 0.13	4.35E-06	UK-FCI	
TIGRP2P100978	7	58,825,151	0.15	0.63 ± 0.13	4.35E-06	UK-FCI	
BICF2G630561837	7	58,845,222	0.15	0.61 ± 0.13	7.57E-06	UK-FCI	
BICF2G630562441	7	59,795,168	0.15	0.59 ± 0.13	1.35E-05	UK-FCI	
BICF2P321938	8	65,857,629	0.33	0.37 ± 0.08	2.59E-06	SWE-FCI	
BICF2S23027935	9	31,300,189	0.49	0.34 ± 0.08	1.42E-05	FIN-FCI	***ANKFN1****, NOG*
BICF2P742007	9	31,387,114	0.44	0.36 ± 0.08	7.51E-06	FIN-FCI	*ANKFN1, NOG*
BICF2G630834826	9	31,477,907	0.45	0.34 ± 0.08	1.52E-05	FIN-FCI	*ANKFN1, NOG, C17orf67, DGKE*
BICF2G630835183	9	32,155,751	0.49	0.35 ± 0.08	9.91E-06	FIN-FCI	
BICF2G630835188	9	32,166,146	0.48	0.35 ± 0.08	6.99E-06	FIN-FCI	
BICF2G630835202	9	32,181,375	0.49	0.34 ± 0.08	1.35E-05	FIN-FCI	
BICF2G630835214	9	32,190,814	0.49	0.34 ± 0.08	1.48E-05	FIN-FCI	
BICF2G630835223	9	32,271,830	0.47	0.36 ± 0.08	7.23E-06	FIN-FCI	*CCDC182*
BICF2G630836291	9	34,689,620	0.38	0.35 ± 0.08	1.59E-05	FIN-FCI	***MED13****, INTS2, BRIP1*
BICF2G630836291	9	34,689,620	0.32	0.28 ± 0.06	3.13E-06	Meta-analysis-FCI	***MED13****, INTS2, BRIP1*
BICF2G630836293	9	34,700,358	0.37	0.35 ± 0.08	1.37E-05	FIN-FCI	***MED13****, INTS2, BRIP1*
BICF2G630836293	9	34,700,358	0.32	0.28 ± 0.06	2.57E-06	Meta-analysis-FCI	***MED13****, INTS2, BRIP1*
BICF2G630836294	9	34,723,827	0.37	0.36 ± 0.08	1.05E-05	FIN-FCI	***MED13****, INTS2, BRIP1*
BICF2G630836294	9	34,723,827	0.32	0.28 ± 0.06	2.39E-06	Meta-analysis-FCI	***MED13****, INTS2, BRIP1*
BICF2G630837240	9	36,579,921	0.46	−0.35 ± 0.08	9.21E-06	FIN-FCI	*ZNHIT3, MYO19, PIGW, GGNBP2, DHRS11, MRM1, LHX1, AATF*
BICF2G630837405	9	36,837,067	0.46	0.36 ± 0.08	3.63E-06	FIN-FCI	*LHX1,****AATF****, ACACA*
BICF2G630442661	15	12,679,667	0.15	−0.35 ± 0.08	8.81E-06	Meta-analysis-FCI	*SPATA6, SLC5A9, TRABD2B*
BICF2P110724	21	39,241,001	0.11	0.85 ± 0.16	4.00E-07*	UK-FCI	*SOX6, ENSCAFG00000030220, PLEKHA7*
BICF2P689487	21	39,270,633	0.11	0.86 ± 0.16	3.81E-07*	UK-FCI	*SOX6, ENSCAFG00000030220, PLEKHA7*
TIGRP2P285227	21	39,296,148	0.11	0.85 ± 0.16	4.00E-07*	UK-FCI	*SOX6, ENSCAFG00000030220, PLEKHA7*
TIGRP2P285228	21	39,304,525	0.11	0.85 ± 0.16	4.00E-07*	UK-FCI	*SOX6, ENSCAFG00000030220, PLEKHA7*
BICF2P865829	21	39,466,638	0.11	0.83 ± 0.16	3.63E-07*	UK-FCI	*ENSCAFG00000030220,****PLEKHA7***
TIGRP2P333312	25	46,422,704	0.08	−0.27 ± 0.06	1.28E-05	SWE case-control	*AGAP1*

^$^AF; allele frequency for the population the significant/ suggestive SNP was identified in (allele frequency within all populations is given in Table S2)

*Genome-wide significant p-value after Bonferroni correction

^§^Genes located within 200 kb of SNPs. Genes are highlighted in bold if a significant or suggestive SNP was intragenic. Empty entries indicate there are no genes within 200 kb of the SNP

The locations of SNPs with significant and suggestive association were mapped to the CanFam3.1 assembly and an area of 200 kb around these SNPs was scanned for genes, revealing 39 positional candidate genes (6 genes with an intragenic SNP; Table 1). Analysis of these 39 genes in Enrichr revealed that the trait “Body mass index” in the GWAS Catalog 2019 was the fifth most significantly enriched term (adjusted *p*-value = 0.009; genes: *GGNBP2, DHRS11, ZNHIT3, LHX1, PIGW, AATF, MRM1, MYO19, PLEKHA7*, Table S3).

### Comparison of populations

To identify whether there was evidence of common genetic architecture of CHD in the individual GSD populations, we examined the overlap between SNPs identified in GWAS for individual populations (UK, SWE and FIN). There were no overlaps in the top 0.1% SNPs between the three populations, however, in pairwise comparisons of the top 1% SNPs (621 or 622 per population) across populations, there were some overlaps (UK-FIN 6, UK-SWE 8 and SWE-FIN 9 for FCI grade; UK-FIN 11, UK-SWE 6 and SWE-FIN 4 for case-control). Based on hypergeometric tests, these results do not suggest a level of overlap beyond what would be expected by chance (except for the UK-FIN case-control comparison where the probability of 11 or more overlaps occurring by chance is 0.05).

## Discussion

GSDs represent one of the largest purebred dog populations in Europe and as a large-sized breed prone to CHD, long-term routine CHD screens (by various organisations) provide a unique opportunity to analyse the genetic architecture of CHD in genetic association studies. However, the inconsistent CHD scoring systems across countries requires specific strategies to enable joint GWAS. In this study, we combined CHD phenotype and SNP genotype data from two countries (UK and Sweden) with publicly available data from a Finnish GSD population [11] to perform single-population and meta-analysis GWAS for CHD, employing various approaches to define the phenotype. We used these analyses to investigate the performance of combined analyses and to identify novel risk loci for CHD.

### QTLs for CHD

We identified five genome-wide significant and several suggestive associations between different CHD phenotypes and SNPs in GWAS of UK, Swedish and Finnish populations, where the Finnish population GWAS is a repetition of the original GWAS in Mikkola et al. [11] using altered phenotypes. There were no SNPs with significant or suggestive associations that overlapped between the single-population analyses and the top regions were located on different chromosomes for each population (e.g. Chr 1 and 9 for FIN, Chr 7 and 21 for UK). Similarly, comparison with reported associations between SNPs and CHD in two previous studies of German GSD populations (FCI screening scheme using a case-control approach: FCI grades A vs. C-D) also indicated primarily population-specific associations; these were found on Chr 19, 24, 26 and 34 [9] and on Chr 1, 3, 4, 8, 9, 16, 19, 26, and 33 [15]. Most of these regions did not overlap with the regions identified in our study, but a QTL was found by Marschall and Distl [15] on Chr 9 at 37.4 Mb, which may be the same locus as the one identified at 36.6 Mb in our study for the repeated GWAS of the Finnish population (FIN-FCI). The limited overlap between genomic regions identified for populations examined in this study and in the literature suggests a primarily population-specific genetic architecture of CHD. The fact that allele frequencies at significant and suggestive SNPs were similar across populations suggests that the lack of consistency was not due to limited power of the genotype datasets (Table S2). However, the lack of consistency could be an artifact of small sample sizes, especially for the UK population, which decreased the power to detect QTL.

The inconsistency of identified SNPs associated with CHD is also seen in comparisons with studies of other dog breeds. For example in Bernese mountain dogs, SNPs significantly associated with FCI grades were identified on Chr 14 and 37 [8], while in Labrador retriever dogs, Sánchez-Molano et al. [4] identified SNPs with chromosome-wide significance associated with HS on Chr 1, 2, 11, 15, 21 and 23 [4]; and a study on the Norberg angle phenotype in 69 breeds found only one significant SNP on Chr 28 [10]. While we found no signals on most of these chromosomes, we did identify genome-wide significant SNPs for UK FCI on Chr 21 in the same region of the QTL for HS and various other CHD-associated traits reported by Sánchez-Molano et al. [4]. There is however some evidence for a common genetic basis of CHD between dog breeds, as Mikkola et al. [16] demonstrated a significant across-breed association with CHD at four out of 46 risk loci identified in previous studies. As described above, CHD is a complex trait and the growing evidence points to a polygenic architecture. There were a few overlaps between highly significant SNPs in the different populations covered by this study but reviewing this and previously published studies, the identified genomic regions generally differ between populations and breeds and furthermore, a major risk loci for CHD has yet to be identified. If the genetic architecture of CHD is largely population-specific, the benefit of multi-population panels is questionable. In our study, most of the suggestive SNPs identified in the meta-analysis overlapped with suggestive SNPs identified in the Finnish population (Chr 1 and 9). This is not surprising since the number of Finnish GSDs exceeded the sample sizes of the UK and Swedish populations and thus, findings might be biased towards the Finnish population. However, other suggestive associations on Chr 7 and 15 were identified in the meta-analysis case-control and FCI analyses, respectively, but were not picked up in any of the single-population analyses, suggesting that the multi-population approach can in some cases provide increased power for QTL detection.

### Technical implications for single and multi-population approaches

The approach for routine CHD scoring (e.g. FCI grades or BVA/KC scores), and thus the assessed CHD phenotype, depends on the geographical location of the dog population. For multi-population approaches with differing scoring schemes, we suggest the conversion of BVA/KC scores into FCI grades, as previously described in the literature [13]. We tested the conversion of both BVA/KC HS and worst hip score (WHS) into FCI grades and found that the two phenotypes were highly correlated (r = 0.997; only one dog was grouped into a different FCI grade between the two approaches). Therefore, we conclude that both BVA/KC score phenotypes are equally suitable for multi-population GWAS. The GWAS results for UK FCI and UK HS were similar, indicating that the conversion from BVA/KC scores to FCI grades was successful. In addition to the choice of BVA/KC phenotype, the CHD phenotypes can be treated as ordinal or case-control response variables. In this study, we performed single and multi-population (meta-analysis) GWAS for both response variable types, but unlike some previous studies [8, 15, 17], we did not exclude intermediate phenotypes (FCI-B dogs) in order to maximize the sample size. Despite this difference, we were able to replicate the identified regions previously reported in the original GWAS for the Finnish population [11, 17], which indicates that including this category is a valid option.

There was not strong consistency between the analyses of ordinal variables and the case-control approach for significant or suggestive SNPs; aside from suggestive SNPs for the Finnish population on Chr 1, no overlaps were found between the two CHD phenotype approaches. Furthermore, more significant or suggestive SNPs were found using HS or FCI grades than using the case-control classification. In the case of the UK population (the smallest sample), there is a peak on Chr 21 for both FCI grades and the case-control classification, although there was no suggestive association for the latter. The significant region on Chr 7 was only detected for FCI. The selection of extreme phenotypes of a quantitative trait to generate a case-control approach in GWAS has previously been shown to increase the power of association studies [18]. Because we included all individuals and did not focus on the extremes, the case-control approach may have been less informative in our study. We also explored the use of an alternative case-control approach in which dogs with FCI scores A and B were classified as controls (instead of only the ‘A’ dogs). No suggestive associations were found with the meta-analysis but the previously noted regions on Chr 1 and 7 were identified in the Finnish and UK populations, respectively (results not shown). We conclude that neither of the case-control approaches is as sensitive as the approach based directly on FCI scores.

Finally, we compared significant and suggestive SNPs found for single population GWAS with the results from the meta-analysis (multi-population) GWAS. The advantage of using multiple populations has been shown in humans for a number of phenotypes [12]. Those authors hypothesized that genetic loci underlying a specific phenotype were the same across populations but there was heterogeneity in effect size. Consequently, they concluded that incorporating multiple populations in the genetic discovery of complex traits would be beneficial in terms of identifying causal (rare) variants. As discussed above, most suggestive SNPs identified in our meta-analysis overlapped with suggestive SNPs identified in the Finnish population (Chr 1 and 9), presumably due to its larger sample size, but two additional regions were only detected in the meta-analysis analysis.

### Potential candidate genes and mechanisms

The 200 kb regions surrounding suggestive SNPs were investigated for potential candidate genes. In the following discussion, we focus primarily on those in which suggestive or genome-wide significant SNPs are located. The regions identified on Chr 1 and Chr 9 include the NADPH Oxidase 3 (*NOX3*) and Ankyrin Repeat And Fibronectin Type III Domain Containing 1 (*ANKFN1*) genes previously reported in the original GWAS by Mikkola et al. [17].

In addition to these previously highlighted candidates, we also identified a nearby candidate gene on Chr 9 associated with CHD: the Mediator Complex Subunit 13 (*MED13*) gene, which encodes the mediator complex subunit 13 and plays a key role in transcription regulation, harboured six suggestive SNPs for the meta-analysis and repeated Finnish FCI GWAS. In mice, a transcriptional regulatory mechanism for the control of skeletal muscle glucose homeostasis controlled by *MED13* was shown through skeletal muscle-specific deletion of the gene [19]. Furthermore, it was previously reported that hip dysplasia was one of the clinical features of a human patient with *MED13* mutations [20]. Interestingly, targeted sequencing of a 7-Mb region on Chr 9 revealed a variant in *MED13* that segregated between CHD cases and controls in the original analysis in the Finnish population [17]. The functional prediction of the variant showed no significant effect in the original work, however, in our study, using the FCI phenotype for both the Finnish population and in the meta-analysis resulted in the detection of multiple suggestive, intragenic SNPs. The meta-analysis case-control GWAS also revealed a candidate region on Chr 7, with a suggestive SNP within the Vasohibin 2 (*VASH2*) gene. Among other functions, *VASH2* has been reported to be involved in mammary tumour development in dogs [21] and to enhance angiogenesis in mice [22]. In a study on soft tissue gene expression in CHD, the biological function “angiogenesis” was enriched by differentially expressed genes between CHD affected and unaffected dogs [23]. In humans, enhanced angiogenesis is a well-known consequence of osteoarthritis [24]. It is possible that the association with the variant in the *VASH2* gene reflects the osteoarthritis aspect of the FCI score (reflected in the higher grades).

A broader analysis of the genes located within 200 kb of suggestive SNPs revealed that several of them (*GGNBP2, DHRS11, ZNHIT3, LHX1, PIGW, AATF, MRM1, MYO19, PLEKHA7*) were previously associated with body-mass index (BMI) in GWAS studies. Pleckstrin Homology Domain Containing A7 (*PLEKHA7*) is particularly interesting because a genome-wide significant SNP (on Chr 21) for UK FCI was located within this gene. The prevalence of hip dysplasia was previously associated with BMI in dogs [25], suggesting obesity as a possible risk factor for CHD. Thus, it is possible that some of the heritable component of HD is related to BMI. Unfortunately, as we did not have information on body size for all of the dogs, we could not fit this as a fixed effect in our models.

## Conclusions

Despite the efforts from various breeding programs, CHD remains a common disease in dogs with a large impact on animal welfare, but the biological basis is not well-understood. In this study, we confirmed the complex genetic nature of the trait, with multiple loci associated with CHD in German Shepherd dogs and also observed that most associated SNPs are population-specific. However, some genomic regions were only identified in the meta-analysis of three populations, thus indicating that routinely assessed CHD information collected across countries provides an opportunity to increase sample sizes and statistical power for association studies. The lack of standardisation of CHD assessment schemes poses a challenge, but conversion of traits can be utilised to overcome this obstacle. Further investigation into the population-specific nature of CHD will help to uncover the biological basis of this disease and will inform selection schemes.

## Materials and methods

### Genotypes

For the UK and Swedish populations, DNA was extracted from saliva samples collected with Performagene PG-100 swabs (UK dogs) and blood (Swedish dogs). The dogs were genotyped using the Illumina CanineHD Whole-Genome Genotyping BeadChip featuring 172,115 SNPs. Quality control procedures were carried out, as previously described in Friedrich et al. [26]. Filtering was imposed in GenomeStudio version 2.0 for sample call rate > 90%, SNP call rate > 98%, reproducibility (GTS) > 0.6 and low or confounded signal characterised by AB R mean (mean normalized intensity of the AB cluster) > 0.3. SNPs were also filtered using PLINK version 1.9 [27, 28] to remove those with minor allele frequency (MAF) < 0.05 and significant deviations from Hardy-Weinberg equilibrium (HWE) (Bonferroni-corrected *p*-value of 0.05 = 4.5 × 10^–7^), resulting in 78,088 autosomal SNPs. For the meta-analysis, we used publicly available data for a Finnish GSDs population from a study by Mikkola et al. [11]. In their study, DNA was extracted from preserved blood samples and dogs were genotyped with the same genotype array as described above. For quality control, we filtered their data for a sample call rate > 90%, a SNP call rate > 98%, MAF > 0.05 and deviations from HWE in PLINK version 1.9 [27, 28], which resulted in 75,271 autosomal SNPs. As final step, we extracted SNPs that overlapped between the UK, Swedish and Finnish populations (*n* = 62,089), which were used for all subsequent analyses. Most of the SNPs that did not overlap between the two datasets had been removed due to the very stringent filtering for SNP call rate.

### Canine hip dysplasia (CHD) phenotypes

CHD records for genotyped UK dogs were provided by the British Kennel Club (KC) and for Swedish dogs by Svenska Kennelklubben (SKK). GSDs from the UK population were bred by multiple breeders and primarily were pet dogs. All GSDs from the Swedish population were bred within the breeding program of the Swedish Armed Forces (SAF), which was founded in 2004 with the purpose of breeding working dogs. The CHD records for the Finnish dogs we used for the meta-analysis are publicly available from the study of Mikkola et al. [11] and they were chosen based on Finnish Kennel Club data with balanced sampling for the dog’s function (working dogs, show dogs, working and show dogs). For the UK dogs, the hip status was screened by the BVA/KC scheme. In this scheme, determined by the severity of HD-related measurements from normal to severe, aggregated scores for bilateral joints (total hip score; HS) are given from 0 to 106 (0 to 53 for each joint). The Swedish and Finnish dogs were scored according to the five-grade FCI scheme, where the CHD severity of the hip joint is classified into A, B, C, D or E grades. The FCI score was only available for the worst hip joint in the Swedish population, thus for data compatibility, we also used the FCI score for the worst hip joint in the Finnish population in this analysis.

To analyse the performance of different CHD phenotypes for the single and multi-populations GWAS, various approaches were used (Table 2).

**Table 2:** Different phenotypic approaches used to analyse CHD in GWAS

Approach	Phenotype for GWAS
*Single populations*	
UK HS	Total hip score
UK FCI	HS converted to FCI*
UK case-control	Cases (BVA/KC scores > = 11) vs. controls (scores 0–10)
SWE FCI grades	FCI grades
SWE case-control	Cases (FCI grades B-E) vs. controls (FCI grade A)
FIN FCI grades	FCI grades
FIN case-control	Cases (FCI grades B-E) vs. controls (FCI grade A)
*Meta-analysis*	
FCI grades	FCI grades with HS-transformed UK dogs*
Case-control	Cases (FCI grades B-E for SWE and FIN, BVA/KC > =11 for UK) vs. controls (FCI grade A for SWE and FIN, scores 1–10 for UK)

*Conversion from BVA/KC scores to FCI grades: 0–10 = A, 11–25 = B, 26–35 = C, 36–50 = D, 51–106 = E

As a first approach for the single population analysis, HS and FCI-transformed HS as described below (UK population) and FCI grades (Swedish and Finnish populations) were used as the response variables. We repeated the single-population GWAS for the Finnish population because we used a different CHD phenotype (FCI of the worst hip joint only, inclusion of FCI B dogs) in contrast to the original GWAS performed by Mikkola et al. [11]. In addition to HS, dogs in the UK also had scores for their worst hip (WHS). In an initial analysis, we tested both HS and WHS and found a high correlation between the GWAS results (r for *p*-values = 0.93 and r for effect sizes = 0.98). Therefore, in the following, we focus on HS. In a second approach, dogs were classified into cases and controls. Based on a previous comparison of different CHD scoring systems [29], GSDs with a total hip score ≤ 10 (UK population) or grade A (Swedish and Finnish population) were considered as controls. The remaining dogs were treated as cases. In contrast to the methodology described in Mikkola et al. [11], we did not exclude intermediate dogs (FCI-B), as this would have decreased the sample size substantially for the UK population. The number of dogs in the different categories can be found in Table 3.

**Table 3:** Number of dogs in the different CHD phenotype categories

BVA/KC scores	FCI grades	UK	SWE	FIN	Meta-analysis
0–10	Grade A	87	152	358	597
11–25	Grade B	75	128	74	277
26–35	Grade C	6	81	126	213
36–50	Grade D	5	33	162	200
51–106	Grade E	7	8	55	70
In total		180	402	775	1357
# of case/control		93/87	250/152	417/358	760/597

The meta-analyses required alignment of the FCI and BVA/KC scoring schemes. BVA/KC total hip scores for UK dogs were converted into FCI five-level grades following a recommended conversion (0–10 = A, 11–25 = B, 26–35 = C, 36–50 = D, 51–106 = E) [13]. As CHD phenotypes for the meta-analysis GWAS, we again used two approaches: (I) the FCI grade (using the above conversion for UK GSDs) and (II) a grouping into cases and controls as described above (A = controls, B-E = cases). CHD records and genotypes were available for 180 UK, 402 Swedish and 775 Finnish GSDs (1357 in total) (Table 3).

### Analysis of population structure

To identify underlying population structure, principle component analysis (PCA) was performed within and across all three populations using PLINK version 1.9 [27, 28]. A pruned SNP dataset was used to account for linkage disequilibrium as recommended in the PLINK documentation. Therefore, the combined SNP dataset was pruned based on the variance inflation factor with default parameters set by PLINK (windows size = 50, shift steps of SNP numbers = 5, the variance inflation factor threshold = 2), resulting in 5167 SNPs. Then, the PCA was performed using this pruned dataset separately within and across populations.

### Building the genetic model

To build the genetic model, the genetic structure and non-genetic factors were tested for their effect on CHD phenotypes. In addition to the first two principal components (PCs) obtained from the PCA, the following non-genetic factors were tested for each CHD phenotype within populations and for the meta-analysis (across populations): sex, birth year, birth month and age at radiographing. Additionally, for the meta-analyses, ‘population’ was also fitted in the model. All factors were fitted as fixed effects in a linear model in R [30] and backward elimination was implemented using the ‘stepAIC’ function of the R package ‘MASS’ to remove one factor at a time and select the model with the lowest Akaike information criterion (AIC). The final genetic models for the CHD phenotypes are shown in Table 4.

**Table 4:** Fixed effects analysed for their effect on CHD phenotypes. Factors fitted in the final model for the GWAS are indicated by “x”

		Sex	Birth year	Birth month	Age radiographing	PC1	PC2	Population
FCI grade	UK							n.a.
	SWE					x		n.a.
	FIN		x	x	x	x	x	n.a.
	Meta-analysis		x		x	x		x
Cases vs. controls	UK							n.a.
	SWE	x				x		n.a.
	FIN		x	x	x		x	n.a.
	Meta-analysis		x					

n.a. not applicable

### Genome-wide association study (GWAS)

The GWAS to identify associations between markers and HD was performed using GEMMA [31] on the 62,089 common SNPs for the single and meta-analysis approaches. The univariate linear mixed model was fitted as below:

$$ y=1\mu + Xb+ c\beta + Z\alpha +e $$

with the following terms: *y* is a vector of CHD phenotypes (depending on the approach), *μ* is the overall mean, *b* is a vector of fixed effects (as described in Table 4) with *X* as the corresponding incidence matrix, *c* is a vector of filtered SNPs (alleles coded as 0/1) with *β* as the corresponding regression coefficients, *Z* is the incidence matrix for the vector of random polygenic effects, *α*, and *e* is a vector of residuals. The vectors of polygenic effects and residuals follow multivariate normal (MVN) distributions given by $MVN\left(\mathbf{0},{\sigma}_{\boldsymbol{\alpha}}^{\mathbf{2}}\mathbf{G}\right)$ and $MVN\left(\mathbf{0},{\sigma}_{\boldsymbol{e}}^{\mathbf{2}}\mathbf{I}\right),$ respectively, where **G** is the genomic relationship matrix composed of the filtered, common SNPs, **I** is the identity matrix and ${\sigma}_{\boldsymbol{\alpha}}^{\mathbf{2}}$ and ${\sigma}_{\boldsymbol{e}}^{\mathbf{2}}$ are the genetic variance associated with **G** and the environmental variance, respectively.

To correct for multiple testing, the Bonferroni correction was applied to account for the number of tests carried out per marker. Accordingly, genome-wide significant markers had *P*-values < 8.1E-07 (adjusted P-value = 0.05/62,089). Since the Bonferroni correction is very stringent, especially for genetic studies in pedigree dogs, which have high levels of linkage disequilibrium across the genome, we also identified markers with suggestive associations. Markers with suggestive associations were determined by allowing one false-positive per genome scan (adjusted P-value < 1.6E-05 = 1/62,089; suggestive association). To compare the results across datasets, we further examined the overlaps between SNPs identified across analyses.

### Analysis of candidate genes

The locations of SNPs with significant or suggestive association were mapped to the CanFam3.1 assembly. BEDTools [32] was then used to identify potential candidate genes by extracting genes harbouring significant or suggestive SNPs and genes in close proximity (within 200 kb). In a previous study of the UK and Swedish GSDs [26], we determined the size of the region around identified SNPs that should be scanned for candidate genes by calculating the squared correlation (r^2^) between all pairs of SNPs within 10 Mb. Then, the average r^2^ was calculated for bins of increasing distance between SNPs to identify the distance around SNPs at which average r^2^ drops below 0.5. The longest bin for which average r^2^ > 0.5 was 200 kb and thus this distance was chosen as the region around associated SNPs to be investigated. Since this study is based on the same samples, we applied the same 200 kb region criterion. All potential candidate genes were submitted together to Enrichr [33], which is a tool that contains a large collection of diverse gene set libraries and allowed us to map a set of genes for enriched biological processes, candidate pathways and previous GWAS results. In Enrichr, a Fisher’s exact test is performed, which assumes a binomial distribution and independence of the probability of any gene belonging to any set [33].

## Supplementary Information

**Additional file 1: Fig. S1.** Q-Q plots for single-population GWAS and meta-analysis. **Fig. S2.** Manhattan and Q-Q plot for the GWAS on UK THS phenotype. Genome-wide significance level is indicated by the red line and a suggestive association by the blue line. **Table S1.** Suggestive SNPs associated with UK total hip score (HS). **Table S3.** Enriched terms for the set of 39 positional candidate genes in the “GWAS Catalog 2019” library using Enrichr. The enriched terms are ranked according to their *p*-value.

**Additional file 2: Table S2.** Allele frequencies (AF) and *p*-values for all approaches for significant or suggestive significant SNPs within the populations and meta-analysis. The phenotype for which the SNP was significant or suggestive is specified in “Approach”. GWAS were performed for 180 (UK), 402 (Swedish), 775 dogs (Finnish) and 1357 (meta-analysis) dogs, respectively.

## Data Availability

Genotype and phenotype data for the UK dogs is available from the Dryad Digital Repository: doi:10.5061/dryad.h44j0zpkf. The data for the Swedish dogs are restricted by the SAF for reasons of national security.

## References

[1] Breed Statistics | Orthopedic Foundation for Animals | Columbia, MO [Internet]. Orthopedic Foundation for Animals. [cited 2020 Nov 12]. Available from: https://www.ofa.org/diseases/breed-statistics

[2] Mäki K, Janss LLG, Groen AF, Liinamo A-E, Ojala M (2004). An indication of major genes affecting hip and elbow dysplasia in four Finnish dog populations. Heredity..

[3] Zhou Z, Sheng X, Zhang Z, Zhao K, Zhu L, Guo G, et al. Differential genetic regulation of canine hip dysplasia and osteoarthritis. PLOS ONE. 2010;5(10):e13219. 10.1371/journal.pone.0013219 PMC2952589 20949002

[4] Sánchez-Molano E, Woolliams JA, Pong-Wong R, Clements DN, Blott SC, Wiener P (2014). Quantitative trait loci mapping for canine hip dysplasia and its related traits in UK Labrador retrievers. BMC Genomics.

[5] Janutta V, Hamann H, Distl O (2006). Complex segregation analysis of canine hip dysplasia in German shepherd dogs. J Hered.

[6] Zhu L, Zhang Z, Friedenberg S, Jung S-W, Phavaphutanon J, Vernier-Singer M, Corey E, Mateescu R, Dykes N, Sandler J, Acland G, Lust G, Todhunter R (2009). The long (and winding) road to gene discovery for canine hip dysplasia. Vet J.

[7] Wilson B, Nicholas FW, Thomson PC (2011). Selection against canine hip dysplasia: success or failure?. Vet J.

[8] Pfahler S, Distl O. Identification of quantitative trait loci (QTL) for canine hip dysplasia and canine elbow dysplasia in Bernese mountain dogs. PLOS ONE. 2012;7(11):e49782. 10.1371/journal.pone.0049782 PMC3506637 23189162

[9] Fels L, Distl O. Identification and validation of quantitative trait loci (QTL) for canine hip dysplasia (CHD) in German Shepherd Dogs. PLOS ONE. 2014;9(5):e96618. 10.1371/journal.pone.0096618 PMC4011879 24802516

[10] Hayward JJ, Castelhano MG, Oliveira KC, Corey E, Balkman C, Baxter TL, et al. Complex disease and phenotype mapping in the domestic dog. Nat Commun. 2016;7(1):–10460. 10.1038/ncomms10460. 10.1038/ncomms10460 PMC4735900 26795439

[11] Mikkola L, Holopainen S, Pessa-Morikawa T, Lappalainen AK, Hytönen MK, Lohi H, Iivanainen A (2019). Genetic dissection of canine hip dysplasia phenotypes and osteoarthritis reveals three novel loci. BMC Genomics.

[12] Wojcik GL, Graff M, Nishimura KK, Tao R, Haessler J, Gignoux CR, Highland HM, Patel YM, Sorokin EP, Avery CL, Belbin GM, Bien SA, Cheng I, Cullina S, Hodonsky CJ, Hu Y, Huckins LM, Jeff J, Justice AE, Kocarnik JM, Lim U, Lin BM, Lu Y, Nelson SC, Park SSL, Poisner H, Preuss MH, Richard MA, Schurmann C, Setiawan VW, Sockell A, Vahi K, Verbanck M, Vishnu A, Walker RW, Young KL, Zubair N, Acuña-Alonso V, Ambite JL, Barnes KC, Boerwinkle E, Bottinger EP, Bustamante CD, Caberto C, Canizales-Quinteros S, Conomos MP, Deelman E, Do R, Doheny K, Fernández-Rhodes L, Fornage M, Hailu B, Heiss G, Henn BM, Hindorff LA, Jackson RD, Laurie CA, Laurie CC, Li Y, Lin DY, Moreno-Estrada A, Nadkarni G, Norman PJ, Pooler LC, Reiner AP, Romm J, Sabatti C, Sandoval K, Sheng X, Stahl EA, Stram DO, Thornton TA, Wassel CL, Wilkens LR, Winkler CA, Yoneyama S, Buyske S, Haiman CA, Kooperberg C, le Marchand L, Loos RJF, Matise TC, North KE, Peters U, Kenny EE, Carlson CS (2019). Genetic analyses of diverse populations improves discovery for complex traits. Nature..

[13] Verhoeven G, Fortrie R, Ryssen BV, Coopman F (2012). Worldwide screening for canine hip dysplasia: where are we now?. Vet Surg.

[14] Wang S, Friedrich J, Strandberg E, Arvelius P, Wiener P. Methods to Improve Joint Genetic Evaluation of Canine Hip Dysplasia Across BVA/KC and FCI Screening Schemes. Front Vet Sci. 2020;7:386. 10.3389/fvets.2020.00386/full. 10.3389/fvets.2020.00386 PMC7432227 32850996

[15] Marschall Y, Distl O (2007). Mapping quantitative trait loci for canine hip dysplasia in German shepherd dogs. Mamm Genome.

[16] Mikkola L, Kyöstilä K, Donner J, Lappalainen AK, Hytönen MK, Lohi H, Iivanainen A (2021). An across-breed validation study of 46 genetic markers in canine hip dysplasia. BMC Genomics.

[17] Mikkola LI, Holopainen S, Lappalainen AK, Pessa-Morikawa T, Augustine TJP, Arumilli M, et al. Novel protective and risk loci in hip dysplasia in German Shepherds. PLoS Genet. 2019;15(7):e1008197. https://www.ncbi.nlm.nih.gov/pmc/articles/PMC6668854/. 10.1371/journal.pgen.1008197 PMC6668854 31323019

[18] Li Y, Levran O, Kim J, Zhang T, Chen X, Suo C (2019). Extreme sampling design in genetic association mapping of quantitative trait loci using balanced and unbalanced case-control samples. Sci Rep.

[19] Amoasii L, Holland W, Sanchez-Ortiz E, Baskin KK, Pearson M, Burgess SC, Nelson BR, Bassel-Duby R, Olson EN (2016). A MED13-dependent skeletal muscle gene program controls systemic glucose homeostasis and hepatic metabolism. Genes Dev.

[20] Snijders Blok L, Hiatt SM, Bowling KM, Prokop JW, Engel KL, Cochran JN (2018). De novo mutations in MED13, a component of the mediator complex, are associated with a novel neurodevelopmental disorder. Hum Genet.

[21] Hussain S, Saxena S, Shrivastava S, Mohanty AK, Kumar S, Singh RJ, Kumar A, Wani SA, Gandham RK, Kumar N, Sharma AK, Tiwari AK, Singh RK (2018). Gene expression profiling of spontaneously occurring canine mammary tumours: insight into gene networks and pathways linked to cancer pathogenesis. PLoS One.

[22] Kimura H, Miyashita H, Suzuki Y, Kobayashi M, Watanabe K, Sonoda H, Ohta H, Fujiwara T, Shimosegawa T, Sato Y (2009). Distinctive localization and opposed roles of vasohibin-1 and vasohibin-2 in the regulation of angiogenesis. Blood..

[23] Todhunter RJ, Garrison SJ, Jordan J, Hunter L, Castelhano MG, Ash K, Meyers-Wallen V, Krotscheck U, Hayward JJ, Grenier J (2019). Gene expression in hip soft tissues in incipient canine hip dysplasia and osteoarthritis. J Orthop Res.

[24] Mapp PI, Walsh DA (2012). Mechanisms and targets of angiogenesis and nerve growth in osteoarthritis. Nat Rev Rheumatol.

[25] Comhaire FH, Snaps F. Comparison of two canine registry databases on the prevalence of hip dysplasia by breed and the relationship of dysplasia with body weight and height. Am J Vet Res 2008;69(3):330–3, 333, DOI: 10.2460/ajvr.69.3.330. 10.2460/ajvr.69.3.330 18312130

[26] Friedrich J, Strandberg E, Arvelius P, Sánchez-Molano E, Pong-Wong R, Hickey JM (2019). Genetic dissection of complex behaviour traits in German shepherd dogs. Heredity..

[27] Purcell SM, Chang CC. PLINK 1.9. Available from: www.cog-genomics.org/plink/1.9/.

[28] Chang CC, Chow CC, Tellier LC, Vattikuti S, Purcell SM, Lee JJ (2015). Second-generation PLINK: rising to the challenge of larger and richer datasets. Giga Science..

[29] Flückiger M. Scoring radiographs for canine Hip Dysplasia - The big three organisations in the world [Internet]. OrthoVetSuperSite. 2009 [cited 2020 Nov 13]. Available from: https://www.orthovetsupersite.org/abstract/scoring-radiographs-canine-hip-dysplasia-big-three-organisations-world

[30] "R Core Team. R: A language and environment for statistical computing. Vienna: R Foundation for Statistical Computing; 2018. Available online at https://www.R-project.org/.

[31] Zhou X, Stephens M (2012). Genome-wide efficient mixed model analysis for association studies. Nat Genet.

[32] Quinlan AR, Hall IM (2010). BEDTools: a flexible suite of utilities for comparing genomic features. Bioinformatics..

[33] Chen EY, Tan CM, Kou Y, Duan Q, Wang Z, Meirelles GV, Clark NR, Ma’ayan A (2013). Enrichr: interactive and collaborative HTML5 gene list enrichment analysis tool. BMC Bioinformatics.

