# Supplementary material for: Genome-wide association studies for canine hip dysplasia in single and multiple populations – implications and potential novel risk loci
Source: BMC Genomics. 2021 Sep 2;22:636. doi: 10.1186/s12864-021-07945-z (PMC8414704; doi:10.1186/s12864-021-07945-z)
Supplement: Supplementary file 1 — Additional file 1: Fig. S1. Q-Q plots for single-population GWAS and meta-analysis. Fig. S2. Manhattan and Q-Q plot for the GWAS on UK THS phenotype. Genome-wide significance level is indicated by the red line and a suggestive association by the blue line. Table S1. Suggestive SNPs associated with UK total hip score (HS). Table S3. Enriched terms for the set of 39 positional candidate genes in the “GWAS Catalog 2019” library using Enrichr. The enriched terms are ranked according to their p-value. [file 12864_2021_7945_MOESM1_ESM.docx]

# Genome-wide association studies for canine hip dysplasia in single and multiple populations – implications and potential novel risk loci

Shizhi Wang^1^, Erling Strandberg^2^, Per Arvelius^3^, Dylan N Clements^4^, Pamela Wiener^1^, Juliane Friedrich^1*^

^1^ Division of Genetics and Genomics, The Roslin Institute and Royal (Dick) School of Veterinary Studies, University of Edinburgh, Midlothian, EH25 9RG, UK

^2^ Department of Animal Breeding and Genetics, Swedish University of Agricultural Sciences, PO Box 7023, S-750 07 Uppsala, Sweden

^3^ Swedish Armed Forces Dog Training Centre, Box 194, SE-195 24 MÄRSTA, Sweden

^4^ Royal (Dick) School of Veterinary Studies, University of Edinburgh, Midlothian

EH25 9RG, UK

*Corresponding author


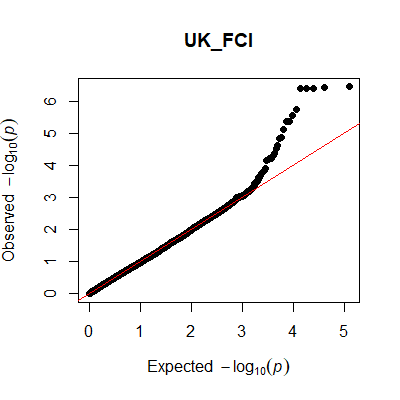

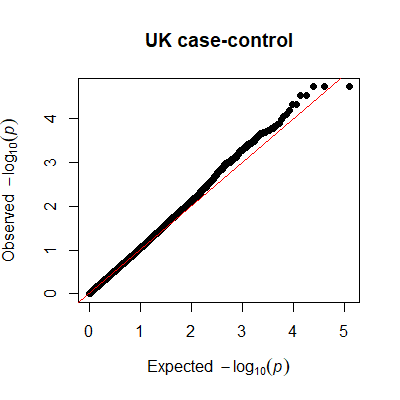

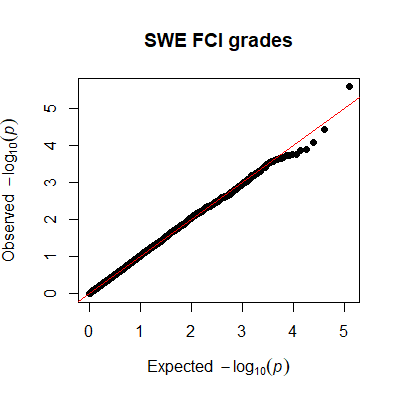

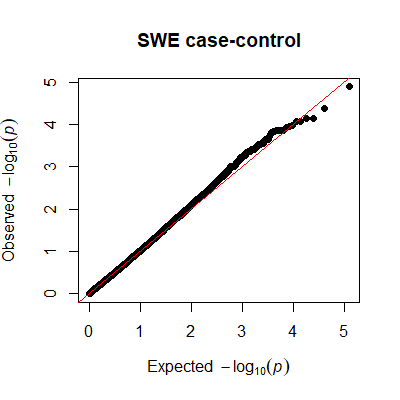

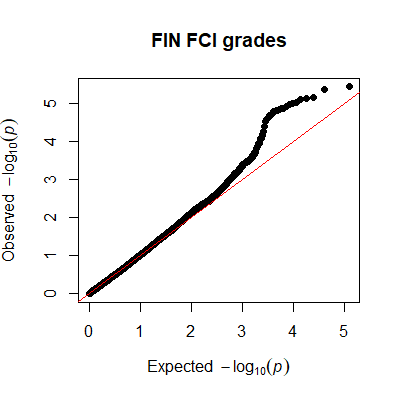

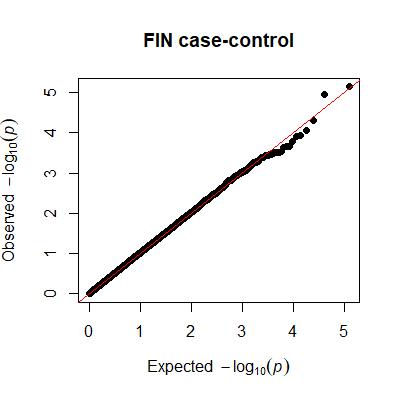

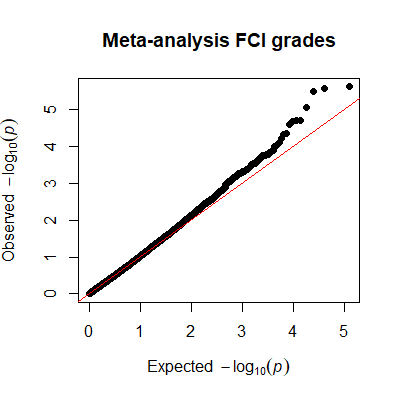

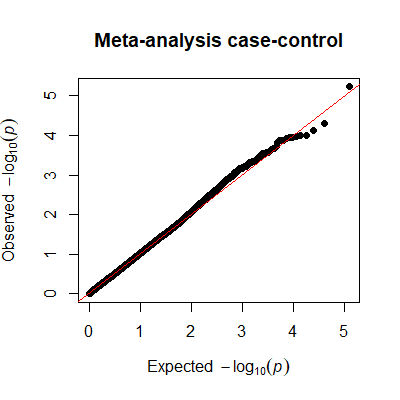


**Figure S1. Q-Q plots for single-population GWAS and meta-analysis**


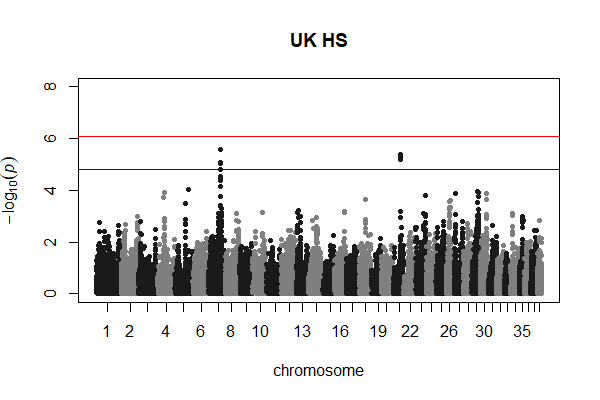

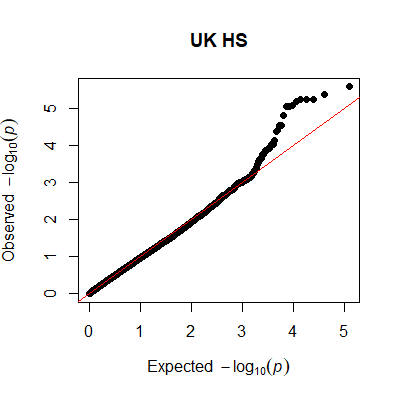


**Figure S2. Manhattan and Q-Q plot for the GWAS on UK THS phenotype.** Genome-wide significance level is indicated by the red line and a suggestive association by the blue line.

**Table S1. Suggestive SNPs associated with UK total hip score (HS)**

| SNP ID | Chr | Pos (bp) | AF^$^ | β±SE | p-value |
| --- | --- | --- | --- | --- | --- |
| BICF2G630561445 | 7 | 58512418 | 0.15 | 8.59 ± 1.87 | 8.31E-06 |
| BICF2G630561553 | 7 | 58588983 | 0.26 | 7.60 ± 1.57 | 2.62E-06 |
| BICF2G630561779 | 7 | 58791059 | 0.15 | 8.76 ± 1.91 | 8.81E-06 |
| TIGRP2P100978 | 7 | 58825151 | 0.15 | 8.76 ± 1.91 | 8.81E-06 |
| BICF2G630561837 | 7 | 58845222 | 0.15 | 8.46 ± 1.91 | 1.58E-05 |
| BICF2P110724 | 21 | 39241001 | 0.11 | 10.81 ± 2.31 | 5.57E-06 |
| BICF2P689487 | 21 | 39270633 | 0.11 | 11.07 ± 2.33 | 4.06E-06 |
| TIGRP2P285227 | 21 | 39296148 | 0.11 | 10.81 ± 2.31 | 5.57E-06 |
| TIGRP2P285228 | 21 | 39304525 | 0.11 | 10.81 ± 2.31 | 5.57E-06 |
| BICF2P865829 | 21 | 39466638 | 0.11 | 10.56 ± 2.27 | 6.36E-06 |

^$^AF; allele frequency

**Table S3. Enriched terms for set of 39 positional candidate genes in the “GWAS Catalog 2019” library using Enrichr.** The enriched terms are ranked according to their p-value.

| Term | Overlap | p-value | Adjusted p-value | Odds Ratio | Combined Score | Genes |
| --- | --- | --- | --- | --- | --- | --- |
| Dimensional psychopathology (Negative) | 4/10 | 0.0000 | 0.0000 | 380 | 7518 | TIAM2; CLDN20; NOX3; TFB1M |
| Dimensional psychopathology (Arousal) | 4/20 | 0.0000 | 0.0000 | 142 | 2373 | TIAM2; CLDN20; NOX3; TFB1M |
| Lipoprotein (a) levels | 3/42 | 0.0001 | 0.0022 | 43 | 405 | TIAM2; NOX3; ARID1B |
| Multiple system atrophy (pathologically confirmed) | 2/16 | 0.0004 | 0.0092 | 77 | 596 | ANKFN1; ARID1B |
| Body mass index | 9/1246 | 0.0005 | 0.0092 | 5 | 34 | GGNBP2; DHRS11; ZNHIT3; LHX1; PIGW; AATF; MRM1; MYO19; PLEKHA7 |
| Nonsyndromic cleft lip with cleft palate | 2/33 | 0.0019 | 0.0273 | 35 | 218 | C17ORF67; NOG |
| Bronchopulmonary dysplasia | 2/39 | 0.0026 | 0.0326 | 29 | 173 | DSC2; DSC3 |
| Ovarian cancer | 2/68 | 0.0078 | 0.0846 | 16 | 79 | BRIP1; ACACA |
| Cannabis dependence | 1/5 | 0.0097 | 0.0851 | 131 | 608 | ANKFN1 |
| Mean arterial pressure x alcohol consumption interaction (2df test) | 2/81 | 0.0109 | 0.0851 | 14 | 61 | SOX6; PLEKHA7 |
| Systolic blood pressure change trajectories | 1/7 | 0.0136 | 0.0851 | 88 | 376 | PLEKHA7 |
| Sensory disturbances after bilateral sagittal split ramus osteotomy | 1/7 | 0.0136 | 0.0851 | 88 | 376 | ARID1B |
| Vein graft stenosis in coronary artery bypass grafting | 1/7 | 0.0136 | 0.0851 | 88 | 376 | ARID1B |
| Diastolic blood pressure x alcohol consumption interaction (2df test) | 2/96 | 0.0151 | 0.0851 | 11 | 48 | SOX6; PLEKHA7 |
| Capecitabine sensitivity | 1/8 | 0.0155 | 0.0851 | 75 | 313 | SOX6 |
| Blood pressure | 2/98 | 0.0157 | 0.0851 | 11 | 46 | SOX6; PLEKHA7 |
| Height | 4/527 | 0.0189 | 0.0872 | 4 | 17 | C17ORF67; DGKE; ANKFN1; NOG |
| Accelerated cognitive decline after conversion of MCI to Alzheimer's disease | 1/10 | 0.0193 | 0.0872 | 58 | 230 | TIAM2 |
| Longevity (90 years and older) | 1/10 | 0.0193 | 0.0872 | 58 | 230 | SOX6 |
| Systolic blood pressure x alcohol consumption interaction (2df test) | 2/115 | 0.0211 | 0.0872 | 9 | 37 | SOX6; PLEKHA7 |
| Glaucoma (primary angle closure) | 1/12 | 0.0232 | 0.0872 | 48 | 180 | PLEKHA7 |
| Eye color | 1/12 | 0.0232 | 0.0872 | 48 | 180 | VASH2 |
| PR segment | 1/12 | 0.0232 | 0.0872 | 48 | 180 | MED13 |
| Objective response to lithium treatment | 1/13 | 0.0251 | 0.0872 | 44 | 161 | MED13 |
| Type 2 diabetes nephropathy | 1/13 | 0.0251 | 0.0872 | 44 | 161 | TRABD2B |
| Celiac disease | 2/130 | 0.0266 | 0.0889 | 8 | 30 | ANKFN1; NOG |
| Heel bone mineral density | 5/898 | 0.0294 | 0.0946 | 3 | 11 | TIAM2; ANKFN1; SOX6; ARID1B; TRABD2B |
| Cotinine glucuronidation | 1/16 | 0.0308 | 0.0956 | 35 | 122 | SOX6 |
| Cleft lip with or without cleft palate | 1/20 | 0.0383 | 0.1149 | 28 | 90 | NOG |
| Adolescent idiopathic scoliosis | 1/23 | 0.0439 | 0.1233 | 24 | 75 | SOX6 |
| Western dietary pattern | 1/23 | 0.0439 | 0.1233 | 24 | 75 | ARID1B |
| Coronary artery calcified atherosclerotic plaque score in type 2 diabetes | 1/25 | 0.0477 | 0.1256 | 22 | 67 | ANKFN1 |
| Alzheimer's disease biomarkers | 1/25 | 0.0477 | 0.1256 | 22 | 67 | ARID1B |
| Urinary albumin excretion (no hypertensive medication) | 1/28 | 0.0532 | 0.1327 | 19 | 57 | TRABD2B |
| Spherical equivalent or myopia (age of diagnosis) | 2/191 | 0.0534 | 0.1327 | 6 | 17 | C17ORF67; NOG |
| Orofacial clefts | 1/32 | 0.0606 | 0.1464 | 17 | 47 | NOG |
| Lumbar spine bone mineral density | 1/36 | 0.0679 | 0.1556 | 15 | 40 | SOX6 |
| Coronary artery calcified atherosclerotic plaque (130 HU threshold) in type 2 diabetes | 1/38 | 0.0716 | 0.1556 | 14 | 37 | AGAP1 |
| Experiencing mood swings | 1/38 | 0.0716 | 0.1556 | 14 | 37 | SOX6 |
| Anti-saccade response | 1/38 | 0.0716 | 0.1556 | 14 | 37 | SOX6 |
| Red cell distribution width | 2/230 | 0.0739 | 0.1569 | 5 | 12 | ZNHIT3; SOX6 |
| Low density lipoprotein cholesterol levels | 1/44 | 0.0824 | 0.1662 | 12 | 30 | ARID1B |
| Systolic blood pressure x alcohol consumption (light vs heavy) interaction (2df test) | 1/44 | 0.0824 | 0.1662 | 12 | 30 | SOX6 |
| Femoral neck bone mineral density | 1/45 | 0.0842 | 0.1662 | 12 | 29 | SOX6 |
| Erectile dysfunction | 1/46 | 0.0860 | 0.1662 | 12 | 29 | AATF |
| Initial pursuit acceleration | 1/51 | 0.0949 | 0.1789 | 10 | 25 | MED13 |
| Protein quantitative trait loci | 1/52 | 0.0966 | 0.1789 | 10 | 24 | DSC3 |
| Bone mineral density (hip) | 1/54 | 0.1002 | 0.1815 | 10 | 23 | SOX6 |
| Diastolic blood pressure (cigarette smoking interaction) | 1/57 | 0.1054 | 0.1865 | 9 | 21 | SOX6 |
| Craniofacial microsomia | 1/58 | 0.1072 | 0.1865 | 9 | 21 | AGAP1 |
| Calcium levels | 1/61 | 0.1124 | 0.1881 | 9 | 19 | ARID1B |
| Hypertension | 1/61 | 0.1124 | 0.1881 | 9 | 19 | SOX6 |
| Urinary albumin excretion | 1/63 | 0.1159 | 0.1902 | 8 | 18 | TRABD2B |
| Systolic blood pressure (cigarette smoking interaction) | 1/66 | 0.1211 | 0.1950 | 8 | 17 | SOX6 |
| Cerebrospinal AB1-42 levels in normal cognition | 1/74 | 0.1347 | 0.2131 | 7 | 14 | BRIP1 |
| Mean corpuscular hemoglobin | 2/336 | 0.1393 | 0.2164 | 3 | 6 | AGAP1; SOX6 |
| Mean corpuscular volume | 2/365 | 0.1588 | 0.2397 | 3 | 5 | AGAP1; SOX6 |
| Bone mineral density | 1/89 | 0.1598 | 0.2397 | 6 | 11 | SOX6 |
| Neurociticism | 1/97 | 0.1729 | 0.2549 | 5 | 10 | SOX6 |
| Diastolic blood pressure x smoking status (current vs non-current) interaction (2df test) | 1/109 | 0.1921 | 0.2763 | 5 | 8 | ARID1B |
| Mean arterial pressure | 1/110 | 0.1937 | 0.2763 | 5 | 8 | PLEKHA7 |
| Coronary artery disease | 2/433 | 0.2065 | 0.2898 | 2 | 4 | SOX6; PLEKHA7 |
| Pancreatic cancer | 1/130 | 0.2247 | 0.3104 | 4 | 6 | ARID1B |
| Neuroticism | 1/142 | 0.2428 | 0.3301 | 4 | 5 | SOX6 |
| Alcoholic chronic pancreatitis | 1/145 | 0.2473 | 0.3310 | 4 | 5 | TRABD2B |
| Lung cancer in ever smokers | 1/153 | 0.2590 | 0.3414 | 3 | 5 | NOX3 |
| Blond vs. brown/black hair color | 1/160 | 0.2692 | 0.3495 | 3 | 4 | SOX6 |
| Reticulocyte count | 1/171 | 0.2848 | 0.3507 | 3 | 4 | SOX6 |
| Allergic disease (asthma, hay fever or eczema) | 1/172 | 0.2862 | 0.3507 | 3 | 4 | ARID1B |
| High light scatter reticulocyte count | 1/172 | 0.2862 | 0.3507 | 3 | 4 | SOX6 |
| High light scatter reticulocyte percentage of red cells | 1/172 | 0.2862 | 0.3507 | 3 | 4 | SOX6 |
| Chronic inflammatory diseases (pleiotropy) | 1/177 | 0.2932 | 0.3529 | 3 | 4 | C17ORF67 |
| Reticulocyte fraction of red cells | 1/180 | 0.2974 | 0.3529 | 3 | 4 | SOX6 |
| Amyotrophic lateral sclerosis (sporadic) | 1/182 | 0.3001 | 0.3529 | 3 | 3 | NOG |
| Macular thickness | 1/191 | 0.3124 | 0.3624 | 3 | 3 | PLEKHA7 |
| Crohn's disease | 2/613 | 0.3368 | 0.3845 | 2 | 2 | C17ORF67; DGKE |
| Menarche (age at onset) | 1/212 | 0.3403 | 0.3845 | 2 | 3 | AGAP1 |
| Diastolic blood pressure | 2/646 | 0.3604 | 0.4020 | 2 | 2 | SOX6; PLEKHA7 |
| Systolic blood pressure | 2/657 | 0.3682 | 0.4055 | 2 | 2 | SOX6; PLEKHA7 |
| Multiple sclerosis | 1/244 | 0.3807 | 0.4140 | 2 | 2 | DSC1 |
| Obesity-related traits | 2/804 | 0.4686 | 0.5033 | 1 | 1 | AATF; DSC3 |
| Intelligence (MTAG) | 1/366 | 0.5137 | 0.5451 | 1 | 1 | GGNBP2 |
| Type 2 diabetes | 1/397 | 0.5428 | 0.5660 | 1 | 1 | ARID1B |
| Intraocular pressure | 1/401 | 0.5465 | 0.5660 | 1 | 1 | PLEKHA7 |
| Pulse pressure | 1/567 | 0.6746 | 0.6905 | 1 | 0 | ARID1B |
| Blood protein levels | 3/2091 | 0.7893 | 0.7985 | 1 | 0 | NOG; PLEKHA7; DSC2 |
| Schizophrenia | 1/882 | 0.8281 | 0.8281 | 1 | 0 | CCDC182 |
